# Real-world treatment patterns for patients receiving second-line and third-line treatment for advanced non-small cell lung cancer: A systematic review of recently published studies

**DOI:** 10.1371/journal.pone.0175679

**Published:** 2017-04-14

**Authors:** Jessica Davies, Manali Patel, Cesare Gridelli, Filippo de Marinis, Daniel Waterkamp, Margaret E. McCusker

**Affiliations:** 1 F. Hoffmann-La Roche Ltd, Welwyn Garden City, United Kingdom; 2 Division of Oncology, Stanford University School of Medicine, Stanford, California, United States of America; 3 Division of Medical Oncology, “S.G. Moscati” Hospital, Avellino, Italy; 4 Division of Thoracic Oncology, European Institute of Oncology (IEO), Milan, Italy; 5 Diagnostics Information Solutions, F. Hoffmann-La Roche AG, Pleasanton, California, United States of America; University of South Alabama Mitchell Cancer Institute, UNITED STATES

## Abstract

Most patients with advanced non-small cell lung cancer (NSCLC) have a poor prognosis and receive limited benefit from conventional treatments, especially in later lines of therapy. In recent years, several novel therapies have been approved for second- and third-line treatment of advanced NSCLC. In light of these approvals, it is valuable to understand the uptake of these new treatments in routine clinical practice and their impact on patient care. A systematic literature search was conducted in multiple scientific databases to identify observational cohort studies published between January 2010 and March 2017 that described second- or third-line treatment patterns and clinical outcomes in patients with advanced NSCLC. A qualitative data synthesis was performed because a meta-analysis was not possible due to the heterogeneity of the study populations. A total of 12 different study cohorts in 15 articles were identified. In these cohorts, single-agent chemotherapy was the most commonly administered treatment in both the second- and third-line settings. In the 5 studies that described survival from the time of second-line treatment initiation, median overall survival ranged from 4.6 months (95% CI, 3.8–5.7) to 12.8 months (95% CI, 10.7–14.5). There was limited information on the use of biomarker-directed therapy in these patient populations. This systematic literature review offers insights into the adoption of novel therapies into routine clinical practice for second- and third-line treatment of patients with advanced NSCLC. This information provides a valuable real-world context for the impact of recently approved treatments for advanced NSCLC.

## Introduction

### Brief background on NSCLC

Globally, lung cancer is the second most common newly diagnosed cancer and the leading cause of cancer death. In 2012, there were an estimated 1.8 million new lung cancer cases and almost 1.6 million deaths [1]. Non-small cell lung cancer (NSCLC) accounts for approximately 83% of all newly diagnosed lung cancers, and most patients (70%) are diagnosed with advanced disease [2]. Despite treatment advancements, overall survival remains poor with 5-year survival estimates globally ranging from 10% to 20% [3–5].

Lung cancer imposes a substantial economic and societal burden [6]. In Europe, lung cancer–related premature mortality cost an estimated €17 billion in 2008 [7]. In the United States, annual lung cancer treatment expenditures were estimated at $13.1 billion USD in 2014 and lost productivity due to premature lung cancer deaths was an additional estimated $36.1 billion USD [2, 8, 9].

### Rationale

Systemic therapy can provide a meaningful clinical benefit for patients with advanced NSCLC, and several new therapies have been approved since 2010 [10]. However, there is a paucity of published information describing how these therapies are used in real-world clinical practice, especially for second and later lines of therapy. Increased understanding of how these treatments are used in routine clinical practice and the associated clinical outcomes may provide insights into how these therapies benefit patients, inform strategies that support development of new therapies, and ultimately decrease the global economic and societal burden of NSCLC.

### Objectives

The primary objective of this study was to describe treatment patterns and survival outcomes among patients who received second-line treatment for advanced NSCLC in routine clinical practice. The study also describes treatment patterns and available information on survival for the subset of patients who received third-line treatment.

### Approach

To achieve these objectives, we conducted a systematic literature review and qualitative evidence synthesis of observational studies.

## Methods

### Literature search strategy

This systematic review was conducted in accordance with Preferred Reporting Items for Systematic Reviews and Meta-Analyses (PRISMA) guidelines [11]. A review protocol does not exist. A tiered search string that included a combination of keywords and medical subject headings (MeSH; S1 Table) was used to search the following databases: BIOSIS Previews, Current Contents Search, Embase, Gale Group PROMT, International Pharmaceutical Abstracts, Medline, and SciSearch.

### Study selection criteria

Observational cohort studies that included detailed information on second- and third-line treatment patterns in patients with advanced NSCLC were eligible for inclusion. Only English-language studies published in peer-review journals between January 1, 2010, and March 1, 2017, were considered. This date range was intended to capture current real-world data on advanced NSCLC treatment patterns in order to account for changes in treatment guidelines related to newly approved therapies and biomarker-guided treatment decisions. Studies that described only one type of therapy, case reports, clinical trials, conference abstracts, and Delphi panels were excluded. Additionally, studies that introduced selection bias with patient selection criteria like requiring platinum-based chemotherapy or a specific mutation were excluded since the intent of the qualitative review was to describe the treatment patterns in a broad, unselected patient population.

### Study selection process

Article titles and abstracts were initially screened by one reviewer (JD) to identify articles that potentially fulfilled the study selection criteria. Full-text articles were independently evaluated for inclusion by 2 reviewers (JD and MM). Additional publications were identified through examination of references cited by the included publications and were included if they also fulfilled the selection criteria.

### Data extraction

Two independent reviewers extracted key information from each publication, including study location, time period, methods, patient and tumor characteristics, treatment regimens by line of therapy, survival outcomes, response rates, and biomarker testing information. If multiple publications described a single study, the extracted data were combined, and each publication was referenced to reflect this circumstance. Discrepancies during study data extraction were resolved by consensus between the reviewers with reference to the articles. If data differed between 2 publications describing the same study, data from the more recent publication were used.

### Main study measures

The main study measures included the proportion of treatment regimens used in second- and third-line advanced NSCLC treatment and overall treatment rates in first-, second-, and third-line therapy. Treatment lines were defined based on the reporting authors’ definitions. Single-agent chemotherapies included systemic anti-cancer therapies other than targeted therapies that were prescribed as monotherapy. Combination chemotherapies comprised 2 or more systemic anti-cancer therapies, including both chemotherapies and targeted therapies. Single-agent targeted therapies included any targeted therapy prescribed as monotherapy. Investigational drugs included any drugs administered as part of a clinical trial. In studies that did not explicitly report treatment proportions among patients receiving second- or third-line treatment, the proportion was estimated based on the number of patients receiving a specific treatment regimen and the total number of patients receiving second- or third-line treatment. Survival outcomes were reported based on the original definitions described in each study.

### Assessment of bias

Risk of bias and methodological quality of the studies were assessed using the 2013 RTI framework created for the Agency for Healthcare Research and Quality for observational studies [12]. Two authors independently performed this assessment (S2 Table).

## Results

**Fig 1:**
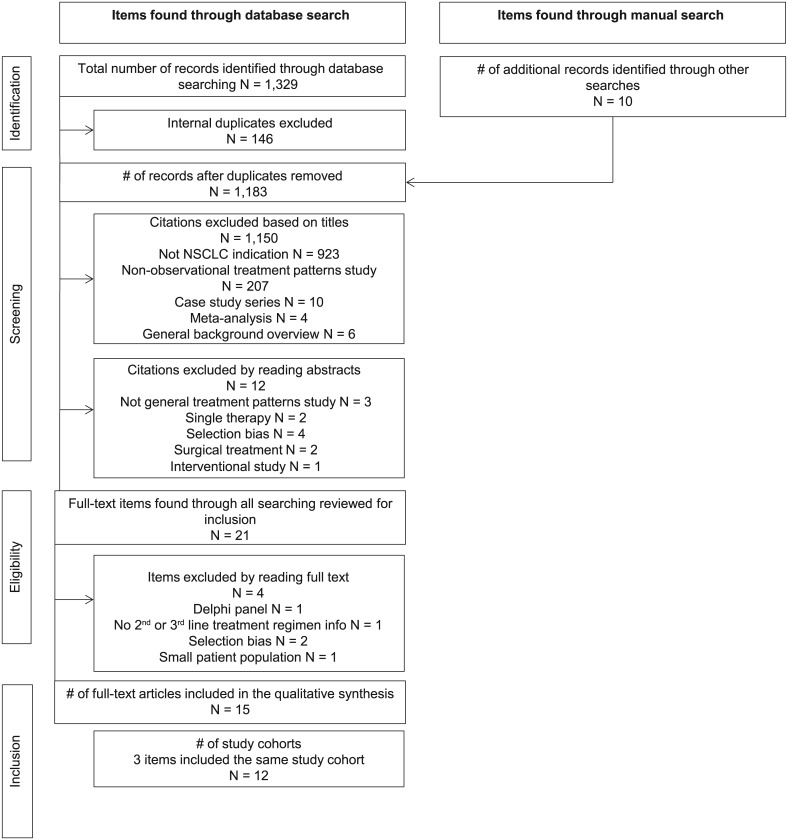
Preferred Reporting Items for Systematic Reviews and Meta-Analyses (PRISMA) flow diagram of studies identified from the systematic literature search [11].

The literature search yielded 1,329 citations, and 10 additional citations were identified through examination of references cited by the included publications (Fig 1). After applying the selection criteria, 15 articles describing 12 different study cohorts were included in the qualitative data synthesis. Of the 12 study cohorts, 7 were retrospective medical record reviews or database analyses and 5 were prospective single-center or multicenter cohorts (Table 1). The study cohorts included patients from Europe (7), North America (3), Asia (1) and South America (1). In general, there was minimal risk of bias within the studies, although several studies had issues that could lead to confounding of the reported outcomes, either due to the length of time during which the study was conducted or missing information on key study variables such as histology or use of oral therapies. Overall, the study results were judged as believable after accounting for limitations.

**Table 1:** Studies evaluating treatment patterns in patients with advanced non-small cell lung cancer by country.

Country	Setting (enrollment period)	Study Population (n)	Selection Criteria	Advanced NSCLC Stage Distribution	Median Patient Age, years (range)	Male (%)	Never Smoker	NSCLC Histology	Definition of Second- and Third-Line Treatment	Study Aims
Brazil [23]	2 cancer research and treatment centers in Sao Paolo (1990–2008)	Adults with cytological or histological diagnosis of stage IV NSCLC (2,673)	1. Cytological or histological diagnosis of stage IV NSCLC 2. Admitted for cancer management according to clinical practice guidelines of each institution	IV: 100%	63 (24–89)	69%	NR	Adenocarcinoma 33% Squamous 31% Large cell 27% NSCLC NOS 10%	Systemic treatment administered after discontinuing first-line chemotherapy, either for intolerance or for progressive or recurrent disease	1. Evaluate patient characteristics of patients with NSCLC 2. Evaluate outcomes of treatment for metastatic disease, with emphasis on second- and third-line chemotherapy
Canada [20]	Linked healthcare databases in Ontario Province (2005–2009)	All patients diagnosed with stage IV NSCLC (8,113)	1. Stage IV NSCLC 2. Diagnosed pre-mortem	IV: 100%	68 (28–96)	54%	NR	Adenocarcinoma 39% Squamous 14% Other 4% NSCLC NOS 43%	Cancer Care Ontario Activity Level Reporting and New Drug Funding Program databases included treatment records with line of therapy documented	Examine practice patterns with respect to systemic treatment, survival outcomes, and changes in chemotherapy costs over time
Europe: Finland, Netherlands, Germany, Portugal, UK [14]	Routine clinical practice in 5 countries (April 2003-September 2004)	Chemonaive adults aged ≥18 years with confirmed stage IIIB or IV NSCLC not participating in a clinical trial (975)	1. Chemo-naive patients aged ≥18 years 2. Not participating in a clinical trial 3. Provided written informed consent to participate	IIIB: 35% IV: 65%	65 (32–90)	71%	NR	NR	Treatment lines were documented by study physicians	1. Investigate potential associations between first-line chemotherapy, general patient and disease characteristics, and outcomes in Europe 2. Evaluate the effect of non–treatment-related factors on outcome in the setting of routine medical care in Europe
Europe and South America: France, Germany, Portugal, Finland, Denmark, UK, Sweden, Netherlands, Israel, Romania, Peru [18]	129 physician practices routinely involved in NSCLC treatment in 11 countries (October 2006-January 2008)	Adults 18+ years with advanced NSCLC who had progressed after first-line chemotherapy and who were about to start second-line treatment (1,013)	1. Initiation of second-line treatment on, after, or not more than 30 days before date of informed consent 2. Not participating in a clinical trial 3. Provided written informed consent to participate	IIIB: 17% IV: 78% Unknown: 5%	63 (32–86)	69%	15%	Adenocarcinoma 52% Squamous 24% Large cell 8% Other 16%	Patients were enrolled around the time of second-line chemotherapy initiation; data were provided by study physicians	1. Assess choice of and time from initiation of second-line treatment to treatment discontinuation in patients with NSCLC 2. Assess reasons for treatment discontinuation 3. Evaluate impact of treatment discontinuation on patient outcomes, including OS and PFS
France [21]	Bas-Rhin population-based cancer registry (1998–2005)	Patients newly diagnosed with stage IIIB (wet) or IV NSCLC and treated in the Bas-Rhin medical network (1,047)	Newly diagnosed with stage IIIB or IV NSCLC Diagnosis not declared on the date of or after death Complete medical record Treated at the Bas-Rhin network	IIIB: 9% IV: 92%	65	75%	13%	Adenocarcinoma 49% Squamous 35% Large cell 13% Other 3%	Treatment data were collected during the course of clinical care	Analyze the management and outcome of NSCLC patients from the department of Bas-Rhin
Germany [24]	Speciality hospital near Munich (January 2003-July 2007)	Treatment-naive patients with histologically confirmed stage IIIB and IV NSCLC (406)	1. Histologically confirmed primary NSCLC 2. Admitted to hospital for diagnostics and/or therapy	IIIB: 16% IV: 84%	65 (57–72)	63%	25%	Adenocarcinoma 59% Squamous 27% Large cell <1% Other 14%	Treatment data were collected during the course of clinical care	1. Present the “real-life conditions” of patients with advanced NSCLC, with a focus on the sequence of different lines of therapy 2. Give information on the complete course of radiotherapy, surgery, and systemic therapy 3. Identify possible prognostic factors for disease control, PFS, and OS for first 3 lines of systemic therapy
Germany [19]	Single academic institution in Heidelberg (January 2004-December 2006)	Patients with stage IV NSCLC (493)	1. No second primary cancers 2. Patients consented in writing for analyses of their data	IIIB: 4% IV: 96%	62 (34–86)	67%	7.50%	Adenocarcinoma 59% Squamous 19% Large cell 22%	Treatment data were collected from medical records of patients treated at the institution	1. Identify patients most likely to benefit from subsequent lines of systemic therapy 2. Understand how clinical study results translate into clinical practice
Italy [15]	60 oncology and pneumology centers in Italy (July 2011-January 2012)	Adults aged 18+ years with stage IIIB or IV NSCLC with disease progression after first-line treatment within 6 months prior to study enrollment (603)	1. ≥18 years of age 2. Histological or cytological stage IIIB-IV NSCLC diagnosis 3. Confirmed disease progression after first-line treatment within 6 months before study enrollment 4. Provided written informed consent to participate	IIIB: 25% IV: 75%	65 (28–84)	70%	25%	Adenocarcinoma 72% Squamous 17% Large cell 2% NSCLC NOS 4% Other 5%	Second-line treatment defined by the clinician as any chemotherapy and/or targeted therapy administered according to routine clinical practice or within a clinical trial subsequent to first-line progression	1. Describe second-line treatment in the clinical setting 2. Describe clinical practice related to biomarker identification in terms of execution, results, and patient features 3. Describe outcome for patients after second-line NSCLC treatment in terms of the proportion of patients who received third-line treatment according to routine clinical practice
Italy [17]	74 oncology and pneumology centers in Italy (January 2007-March 2008)	Adults age 18+ years with newly diagnosed inoperable stage IIIB or IV NSCLC (987)	1. ≥18 years of age 2. Newly diagnosed inoperable NSCLC stage IIIB (T4 with pleural effusion and/or N3 metastatic supraclavicular lymph nodes) or IV 3. No concomitant secondary tumors 4. Provided written informed consent to participate	IIIB: 22% IV: 78%	66 (35–86)	75%	18%	Adenocarcinoma 44% Squamous 28% Large cell 4% NSCLC NOS 24%	Second- and third-line medical treatments defined as any chemotherapy or targeted therapy previously not administered	1. Describe the evolving approaches of the Italian oncologists in the treatment of advanced NSCLC
Japan [13]	Single academic institution in Tokyo (July 2002-June 2006)	Patients with histologically or cytologically proven, unresectable stage IIIB or IV or recurrent NSCLC who had received first-line chemotherapy (599)	1. Histologically or cytologically proven unresectable stage IIIB to IV or recurrent NSCLC 2. Received first-line chemotherapy at the institute	IIIB: 22% IV: 55% Recurrent: 23%	62 (31–81) for patients who received first or second line; 59 (26–76) for patients who received ≥third line	65%	34%	Adenocarcinoma 77% Squamous 12% Large cell 4% NSCLC NOS 7% Other <1%	Treatment data were collected from medical records of patients treated at the institution	Describe use of and outcomes for third- and fourth-line chemotherapy for NSCLC after approval of gefitinib in Japan in 2002
United States [27]	Network of 1200 community-based oncology practices (January 2007-June 2011)	Adults aged 18+ years with stage IIIB or IV non-squamous NSCLC who initiated second-line chemotherapy (1,168)	1. Receipt of care at a site utilizing the full iKM EHR capabilities at time of treatment 2. ≥2 visits within the study period 3. ≥18 years at date of initiating second-line therapy 4. ECOG performance status <3 at date of initiating second-line therapy 5. Not enrolled in a clinical trial 6. No other major cancers	IIIB: 17% IV: 83%	NR (59% ≥65)	54%	11%	Non-squamous 100%	Treatment data were collected from the EHR for patients treated by clinicians in the US Oncology network of community-based oncologists	Evaluate treatment patterns, NSCLC biomarker testing rates, clinical outcome, and hospitalization among patients receiving second-line treatment for advanced NSCLC in a US community-based setting
United States [26]	SEER-Medicare linked healthcare databases (2001–2009)	Patients diagnosed with stage IIIB or IV squamous NSCLC (17,133)	1. Primary stage IIIB with pleural effusion (2004–2009) or stage IV squamous NSCLC (2001–2009) or initial diagnosis at an earlier disease stage with later development of metastases 2. No other primary cancers 3. Aged >65 years 4. Continuous enrollment in Medicare Parts A and B for 6 months before to end of study or death	IIIB: 17% IV: 83%	75.3 (mean)	62%	NR	Squamous 100%	Systemic therapy regimens and cycles were defined using previously published methods that define algorithms based on procedure and diagnostic codes	Assess demographic and clinical characteristics, OS by treatment status, treatment patterns, common systemic treatment regimens used in different therapy lines, and healthcare resource use and costs among patients with metastatic squamous NSCLC enrolled in the US Medicare system

### Patient characteristics

Median age at advanced NSCLC diagnosis ranged from 59 to 68 years in 10 studies that reported this information (Table 1) [13–25]. Males comprised a larger proportion of the study population in all cohorts. There were 2 studies that included only patients with a single NSCLC histology—one with only squamous patients, and one with only non-squamous patients [26, 27]. Among the other studies, adenocarcinoma was the most common NSCLC histology, with the proportion of adenocarcinoma patients ranging from 33% in Brazil to 77% in Japan [13, 23]. Two studies enrolled only stage IV patients [20, 23]. The proportion of patients who never smoked was highest in the Japanese cohort [13,]. Five studies reported performance status after first-line therapy or at initiation of second-line therapy, with most patients having a Karnofsky Performance Status of 70 or better or an Eastern Cooperative Oncology Group score of 0 or 1 [15, 16, 18, 21, 24, 27].

### Treatment patterns

#### Second-line treatment

**Fig 2:**
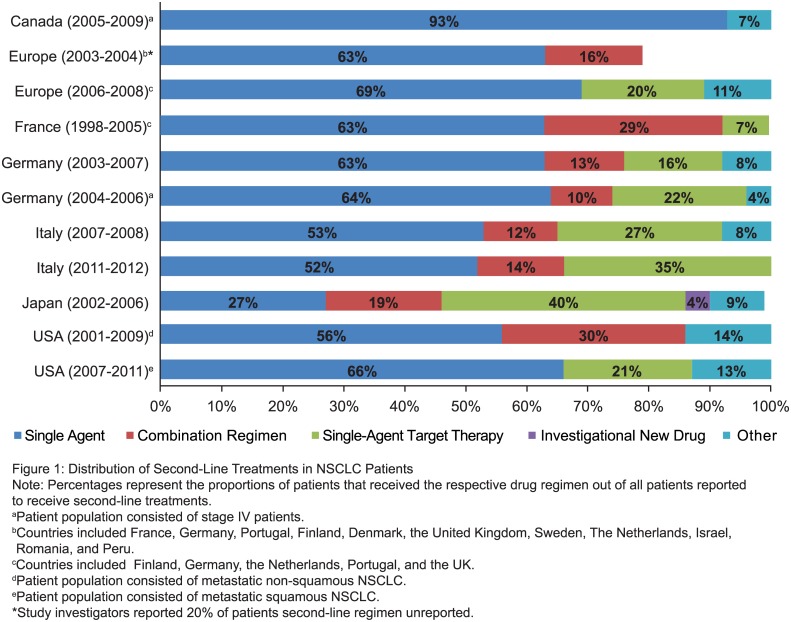
Second-line systemic regimen composition by country and time period. Note: Percentages represent the proportions of patients who received the respective drug regimen out of all patients reported to receive second-line treatments. ^a^ Per study investigators, 20% of patients’ second-line regimens were unreported. Germany (2004–2006) and Canada (2005–2009): Patient population consisted of stage IV patients. Europe (2003–2004): Countries included France, Germany, Portugal, Finland, Denmark, the United Kingdom, Sweden, the Netherlands, Israel, Romania, and Peru. Europe (2006–2008): Countries included Finland, Germany, the Netherlands, Portugal, and the United Kingdom. South Korea (2003–2008): All patients had platinum-based first-line therapy. United States (2007–2011): Patient population consisted of metastatic non-squamous NSCLC. United States (2001–2009): Patient population consisted of metastatic squamous NSCLC. NSCLC, non-small cell lung cancer.

Fig 2 and Table 2 describe the distribution of second-line treatment regimens in each of the studies. The proportion of patients receiving second-line therapy among the studies varied depending on how the study cohort was selected and what treatments were included. In studies that followed patients from initial NSCLC diagnosis, the proportion of patients who received second-line treatment ranged from 8% in a population-based Canadian study that did not include oral therapies (epidermal growth factor receptor [EGFR] tyrosine kinase inhibitors [TKIs]) to 53% in a German study at a single institution [20, 24, 25].

**Table 2:** Summary of second-line treatment patterns.

Country	Reference	Number of Patients Enrolled	Patients With First-Line Treatment, n (%)^a^	Patients With Second-Line Treatment, n (%)^a^	Overall Second-Line Treatment Regimen Distribution, n (%)^b^	Distribution of Single-Agent Treatments, n (%)^b^	Distribution of Combination Regimens, n (%)^b^	Distribution of Targeted Therapy, n (%)^b^
Brazil^c^	Younes et al, 2011	2673	1548 (58%)	625 (40.4%)	Non-platinum based 95% Platinum based 5%	NR	NR	NR
Canada^c^	Sacher et al, 2015	8113	1944 (24%)	609 (31.3%)	Single agent 93% Other 7%	Docetaxel 52% Pemetrexed 41%	NR	NR
Europe^d^	Bischoff et al, 2010	975	975 (100%)	285 (29.2%)	Single agent 63% Combination regimen 16%	Taxane 120 (42%) Vinorelbine 31 (11%) Gemcitabine 29 (10%)	NR	NR
Europe^e^	Moro-Sibilot et al, 2010; Vergnenegre et al, 2012 (SELECTTIONN)	1013	1013 (100%)	1013 (100%)	Single agent 700 (69%) Targeted therapy 206 (20%) Other regimens 106 (11%)	Pemetrexed 468 (46%) Docetaxel 232 (23%)	NR	Erlotinib 207 (20%)
France^f^	Carpentier, 2016	1,047	863 (82%)	431 (41.2%)	Single agent 273 (64%) Combination regimen 126 (29%) Targeted therapy 32 (7%)	Docetaxel 100 (23%) Pemetrexed 17 (4%) Other 156 (36%)	Cisplatin based: 48 (11%) Carboplatin based: 78 (18%)	Erlotinib or gefitinib 32 (7%)
Germany	Zietemann, 2011; Zietemann, 2010	406	406 (100%)	213 (52.5%)	Single agent 134 (63%) Combination regimen 28 (13%) Targeted therapy 35 (16%) Other regimen 16 (8%)	Docetaxel 66 (31%) Gemcitabine 31 (15%) Vinorelbine 24 (11%) Pemetrexed 13 (6%)	Gemcitabine + mitomycin 10 (5%) Carboplatin + paclitaxel 7 (3%) Carboplatin + vinorelbine 5 (2%) Carboplatin + gemcitabine 3 (1%) Carboplatin + docetaxel 3 (1%)	Erlotinib 20 (9%) Gefitinib 15 (7%)
Germany^c^	Reinmuth et al, 2013	493	352 (71%)	183 (52%)	Single agent 117 (64%) Combination regimen 18 (10%) Targeted therapy 41 (22%) Other 7 (4%)	NR	Platinum based 18 (10%)	EGFR-TKI 41 (22%)
Italy	De Marinis et al, 2014; Gridelli et al, 2014 (LIFE)	541	541 (100%)	464 (85.8%)	Single agent 241 (52%) Combination regimen 63 (14%) Targeted therapy 163 (35%)	Docetaxel 118 (25%) Pemetrexed 68 (15%) Gemcitabine 26 (6%) Vinorelbine 23 (5%) Carboplatin 2 (<1%) Cisplatin 2 (<1%) Paclitaxel 2 (<1%)	Carboplatin + gemcitabine 15 (3%) Cisplatin + pemetrexed 14 (3%) Docetaxel + gemcitabine 9 (2%) Carboplatin + paclitaxel 8 (2%) Carboplatin + pemetrexed 7 (2%) Cisplatin + gemcitabine 5 (1%) Other 5 (1%)	Erlotinib 149 (32%) Gefitinib 9 (2%) Bevacizumab combination 3 (1%) Crizotinib 2 (<1%)
Italy	Gridelli et al, 2011	987	790 (80%)	275 (35%)	Single agent 146 (53%) Combination regimen 34 (12%) Targeted therapy 73 (27%) Clinical trial 22 (8%)	Pemetrexed 56 (20%) Docetaxel 46 (17%) Gemcitabine 19 (7%) Vinorelbine 19 (7%) Cisplatin or carboplatin 3 (1%) Other 3 (1%)	Carboplatin + vinorelbine 6 (2%) Carboplatin + pemetrexed 6 (2%) Carboplatin + gemcitabine 4 (1%) Docetaxel + vinorelbine 2 (1%) Carboplatin + paclitaxel 2 (1%) Cisplatin + docetaxel 2 (1%) Cisplatin + gemcitabine 1 (<1%) Other 6 (2%)	Erlotinib 73 (27%)
Japan	Asahina et al, 2012	599	599 (100%)	415 (69%)	Single agent 114 (27%) Combination regimen 80 (19%) Targeted therapy 167 (40%) Investigational new drug 15 (4%) Other 39 (9%)	Docetaxel 114 (27%)	Carboplatin + paclitaxel 64 (15%) Carboplatin + gemcitabine 16 (4%)	Gefitinib 167 (40%)
United States^g^	Pan et al, 2013	1168	1168 (100%)	1168 (100%)	Single agent 781 (66%) Targeted therapy 241 (21%) Other 146 (13%)	Pemetrexed 635 (54%) Docetaxel 117 (10%) Gemcitabine 29 (2%)	NR	Erlotinib 205 (18%) Bevacizumab combination 36 (3%)
United States^h^	Davis et al, 2015	17,133	7029 (41%)	3405 (20%)	Single agent 1916 (56%) Combination regimen 1012 (30%) Other 477 (14%)	Gemcitabine 550 (16%) Docetaxel 459 (14%) Pemetrexed 376 (11%) Vinorelbine 207 (6%) Paclitaxel 206 (6%) Carboplatin 118 (3%)	Carboplatin + paclitaxel 458 (14%) Carboplatin + gemcitabine 299 (9%) Carboplatin + docetaxel 181 (5%) Gemcitabine + vinorelbine 74 (2%)	NR

Abbreviations: EGFR-TKI, epidermal growth factor receptor tyrosine kinase inhibitor; NR, not reported; NSCLC, non-small cell lung cancer.

^a^Percentage of patients with first-line treatment out of all patients

^b^Percentage of patients with second-line treatment.

^c^Only consisted of patients with stage IV NSCLC.

^d^Countries included France, Germany, Portugal, Finland, Denmark, the United Kingdom, Sweden, the Netherlands, Israel, Romania, and Peru.

^e^Countries included Finland, Germany, the Netherlands, Portugal, and the United Kingdom.

^f^Other includes vinorelbine, gemcitabine, paclitaxel, adriamycin, epirubicin, mitomycin, ifosfamide, VP16, cyclophosphamide, 5-fluroruracil, and topotecan

^g^Only included patients who received planned second-line treatment and had non-squamous histology.

^h^Only included patients who had squamous histology.

In 10 studies that provided detailed information on prescribed treatment regimens, single-agent chemotherapy was the most commonly used second-line treatment regimen except in one study from Japan. In the Japanese study, single-agent targeted therapy was the most commonly prescribed second-line treatment [13]. Docetaxel, pemetrexed, and gemcitabine were among the top 3 most frequently used single-agent chemotherapeutics across the studies [15–22, 24–27]. In Japan gefitinib was the most frequently used second-line single-agent targeted-therapy [13]. Outside of Asia, the most frequently used single-agent targeted therapy was erlotinib [15–19, 22, 24, 25, 27].

Pemetrexed was one of the most commonly prescribed second-line treatments in the studies that included patients treated after its initial approval in 2004 [15–18, 22, 24, 25, 27]. The study conducted in Canada reported that pemetrexed use increased to 70% among treated second-line patients in 2008, when government funding was approved for pemetrexed [20].

Second-line treatment with EGFR-TKIs increased in the European studies after initial approval of erlotinib in 2005 [15–19, 21, 22, 24, 25]. This uptake occurred prior to the widespread introduction of EGFR mutation testing, which was not required at the time of initial erlotinib approval.

Platinum-based doublet chemotherapy was administered in all 7 studies that described the use of second-line combination chemotherapy regimens [13, 15–17, 19, 21, 24–26]. The most frequent use of combination chemotherapy was reported in a study that included only patients with squamous histology [26].

#### Third-line treatment

Nine of the 12 studies reported detailed information on patients who received third-line therapy [15–19, 21–25]. In these studies, approximately 30% of patients received third-line treatment (S1 Table). Single-agent chemotherapy accounted for about 50% of third-line treatments in general, and targeted therapy accounted for almost 40% [15–18, 21, 22, 24, 25]. Docetaxel, gemcitabine, vinorelbine, and pemetrexed were the most frequently administered third-line chemotherapies. Erlotinib was the most common single-agent targeted therapy in the third-line setting in all countries.

#### Other lines of therapy

All 12 studies reported details about the distribution of first-line treatment regimens. The most frequently administered first-line treatment across all countries was platinum-doublet chemotherapy (S3 Table). Three European studies reported use of targeted therapy in the first-line setting. In an Italian cohort, gefitinib, bevacizumab, and erlotinib were administered to 6%, 4%, and 1% of first-line treated patients, respectively [15, 16]. Erlotinib was administered to 3% of first-line patients in a German cohort [24, 25]. Only 4 patients (0.4%) in a French cohort received an EGFR-TKI in first-line therapy [21].

In Japan, fourth-line chemotherapy was reported in 17.7% of the patients who received first-line chemotherapy. The top 3 anti-cancer treatment regimens in the fourth-line setting were single-agent S-1 (22%), docetaxel (21%), and gefitinib (21%) [13]. In Brazil, 2.5% of all patients with stage IV NSCLC (4.3% of the patients who received first-line chemotherapy) received fourth-line chemotherapy [23].

### Survival outcomes

Eleven of 12 studies included in this review presented overall survival (OS) data (Table 3) [13, 15–27]. Five studies reported OS from time of metastatic NSCLC (mNSCLC) diagnosis [19, 20, 21, 23, 26]. Three of these studies reported the median OS among patients with advanced NSCLC who received second-line therapy. Median OS from mNSCLC diagnosis date ranged from 8.7 months among patients who received only first- and second-line (ie, no third-line) treatments in a single-center German cohort to 17 months among patients who received at least second-line treatment in a single-center Brazilian cohort [19, 20, 23].

**Table 3:** Summary of survival outcomes by definition of survival time.

Region/Country	Source	Number of Patients	Study Setting	Study Period	Median OS, months (first line)	Median OS, months (second line)	Median OS, months (third line)	Median OS (BSC only)	OS Definition
**Time from mNSCLC diagnosis to death/study end**									
Brazil	Younes et al, 2011	2673	Single institution	1990–2008	11.0	17.0		4.0	Time from initial mNSCLC diagnosis to date of last consultation or death
Canada	Sacher et al, 2015	8113	Population based	2005–2009	8.2 (7.7–8.6) for patients who received first line only	16.2 (15.1–17.0) for patients who received first and second line		3.3 (3.2–3.4)	Time from date of mNSCLC diagnosis (K-M)
France	Carpentier et al, 2016	1047	Population based	1998–2005	8.3 (7.6–9.1)			1.3 (1.1–1.7)	Time from date of mNSCLC diagnosis to date of death, visit to the medical center, or end of study period (K-M)
Germany	Reinmuth et al, 2013	493	Single institution	2004–2006	2.0	5.3			Time from date of mNSCLC diagnosis (K-M)
United States	Davis et al, 2015	17,133	Population based	2001–2010	8 (for patients who received any cancer-directed treatment)			2.0	Time from date of mNSCLC diagnosis to death or end of study period (K-M)
**Time from start of first-line chemotherapy until death/study end**									
Europe (multiple)	Bischoff et al, 2010 (ACTION study)	975	Provider based	2003–2006	9.3 (8.6–10.3)				Time from start of chemotherapy until death or time of last follow-up
Europe (Italy)	Gridelli et al, 2011 (SUN study)	790	Multiple institutions	2007–2008	9.1 (8.1–10.0)				Time from start of first-line chemotherapy until last day patient was known to be alive
**Time from start of each chemotherapy line until death/study end**									
Japan	Asahina et al, 2012	599	Single institution	2002–2006	15.3 (13.8–16.5)	12.8 (10.7–14.5)	12.0 (9.3–14.2)		Time from first day of each chemotherapy line until death or last day of follow-up period
Germany	Reinmuth et al, 2013	493	Single institution	2004–2006	7.6 (6.8–8.5)	6.2 (5.0–7.4)	5.2 (3.5–7.0)		Time from the beginning of the respective line of systemic therapy
Europe (Germany)	Zietemann, 2010 and 2011	405	Single institution	2003–2008	8.9 (8.2–10.1)	4.6 (3.8–5.7)	3.8 (2.6–5.4)		Time from first day of each chemotherapy line until death or last day of follow-up period; OS given in days in study and converted to months (days/30)
**Time from start of second-line chemotherapy until death/study end**									
Europe (multiple)	SELECTTION study—2010, 2012	1013	Provider based	2006–2008		ACA: 8.1 (6.9–9.0) other NSCLC: 6.2 (5.5–6.8)			Time from start of second-line chemotherapy until death or date of last contact
United States	Pan et al, 2013	1168	Provider based	2007–2011		7.5 (6.6–8.4)			Time from start of second-line chemotherapy until death or date of last follow-up visit (K-M)
**Time from the start of third-line chemotherapy until death/study end**									
France	Carpentier et al, 2016	226	Population based	1998–2005			TKI: 5.9 (4.2–10.1)		Time from initiation of third line treatment to date of death, vital status, or end of study period (K-M)
**No outcome data provided**									
Europe (Italy)	LIFE study—2014, 2014	541	Multiple institutions	2011–2012					No survival reported

Abbreviations: ACA, adenocarcinoma; BSC, best supportive care; K-M, Kaplan-Meier; mNSCLC, metastatic non-small cell lung cancer; NSCLC, non-small cell lung cancer; OS, overall survival; TKI, tyrosine kinase inhibitor.

In 5 studies that described median OS from time of second-line treatment initiation in routine clinical practice, median OS ranged from 4.6 months (95% CI, 3.8–5.7) to 12.8 months (95% CI, 10.7–14.5) [13, 19, 22, 24, 25, 27]. Median OS estimates were close to 8 months in 2 studies that reported this information specifically for patients with adenocarcinoma in the second-line setting [18, 22, 27]. Median OS estimates were reported from the time of third-line treatment initiation in 4 studies and median OS ranged from 3.8 (95% CI, 2.6–5.4) to 12.0 months (95% CI, 9.3–14.2) [13, 24, 25]

### Biomarkers

Two studies reported biomarker testing and treatments prescribed based on biomarker status, one in Italy (LIFE) and one in the US that included only patients with adenocarcinoma (Table 4) [15, 16, 27]. In the US study, EGFR testing frequency increased significantly from 2.3% between 2007 and 2009 to 32% in the first 6 months of 2011 [27]. The LIFE study, which was conducted in 2011–2012, reported that 60% of Italian patients received biomarker testing [15, 16]. In this study, biomarker testing was more frequent among patients who were younger, female, never-smokers, and those with adenocarcinomas.

**Table 4:** Summary of biomarker testing frequency and mutation prevalence by country.

	United States [27]	Italy [15, 16]
Study population	Advanced non-squamous NSCLC patients who initiated second-line treatment	Advanced NSCLC patients with disease progression after first-line treatment
Study enrollment period	2007–2011	2011–2012
Timing of biomarker testing	Before or at initiation of second-line treatment	Between advanced NSCLC diagnosis and baseline study visit
Biomarker testing frequency	N (%)	N (%)
Any biomarker	NR	314 (58%)
EGFR	128 (11%)	311 (57%)
KRAS	40 (3%)	77 (14%)
ALK	28 (2%)	74 (14%)
Biomarker mutation prevalence (among tested patients)		
*EGFR*	24 (19%)	65 (21%)
*KRAS*	8 (20%)	17 (22%)
*ALK*	1 (4%)	17 (23%)

Abbreviations: EGFR, epidermal growth factor receptor; NR, not reported; NSCLC, non-small cell lung cancer.

Both studies provided evidence of biomarker-driven therapy choices within their cohorts. The LIFE study reported that 26 of 37 (70%) patients who received EGFR-TKIs as first-line therapy were known to have EGFR-activating mutations prior to first-line treatment [15, 16]. In the US cohort, 50% of 24 patients with known *EGFR* mutations received erlotinib as second-line treatment compared with 17% of the 89 *EGFR* wild-type patients. The use of erlotinib was significantly more common in patients with *EGFR* mutations compared with *EGFR*-wild type patients (*P* < .001) [27].

## Discussion

The objective of this study was to describe real-world treatment patterns and survival outcomes for patients with advanced NSCLC who received second-line or later treatments, through a review of recently published observational studies. To our knowledge, there are no systematic reviews that summarize this information. By qualitatively reviewing this information from multiple countries, we provide a broad overview of how patients are managed in this setting around the world.

Overall, the retrieved studies showed how newly approved therapies are rapidly integrated into clinical practice, and how clinical practice evolves in response to therapeutic advances. In addition, most studies demonstrated a high level of clinician adherence to international treatment guidelines, such as those from European Society of Medical Oncology and the US National Comprehensive Cancer Network [28, 29]. Among the studies that focused solely on patients with non-squamous NSCLC, single-agent pemetrexed, docetaxel, or EGFR-TKIs were the most common treatment choices, which aligns with current guidelines. However, there were examples of non-adherence to guidelines, namely use of platinum-doublet chemotherapies as second-line treatment. This was highest in the all-squamous cohort, which is consistent with the limited number of approved second-line treatments for this particular subgroup of patients with advanced NSCLC that were available at the time these studies were conducted [26]. Moreover, while specific patient-level information was not available from these studies, it is possible that use of platinum-doublet chemotherapy in the second-line setting could be motivated by oncologists’ perceptions of the superiority of these particular treatment regimens.

Survival after initiation of second-line treatment was reported in most studies, and was generally consistent with outcomes reported from pemetrexed, docetaxel, and erlotinib clinical trials [30–33]. The consistency of survival outcomes suggests that clinical trial results may be generalizable to real-world patient populations. Additionally, the data confirm the limited survival benefit of treatments that were available at the time these studies were conducted. An exception to this was the lower survival among second-line patients in Germany reported by Zietemann and Duell compared with outcomes reported from other European studies with similar outcome definitions [19, 22]. No explicit reason for the lower survival reported in this study was identified based on patient and tumor characteristics, or the study setting. The range of survival outcomes reported by the studies included in this review exemplifies the variation that can occur across multiple studies and may be due to individual-level patient differences. In addition, these survival differences highlight the importance of examining population-level data and outcomes from multiple observational studies to understand the range of outcomes experienced in routine clinical practice and the limited ability to draw conclusions from studies that do not report detailed patient-level data.

In most studies that reported the proportion of patients with advanced NSCLC treated across lines of therapy, between one-third to one-half of those who received first-line treatment also received second-line therapy [14, 16, 20, 21, 23–26]. None of these studies provided information to explain why patients did not receive later lines of therapy. However, Gridelli et al reported that poor performance status and older age were the most common reasons patients did not receive first-line treatment [17]. These reasons, in addition to a poor response to first-line treatment, may be why patients with advanced NSCLC do not receive later lines of therapy. Newer treatments such as immunotherapies may offer these patients an opportunity to receive treatment because of their more favorable toxicity profiles compared with existing treatments [34, 35]. Additional studies that examine why patients are not treated could help identify opportunities to increase the proportion of patients with advanced NSCLC who derive benefit from treatment.

There was limited information reported on biomarker-guided therapy decisions in the studies included in this review, likely due to the time periods covered by the studies. In the studies covering more recent time periods, the rapid uptake seen in the use of targeted therapies and biomarker testing is encouraging. It reveals that biomarker-driven treatment decisions are being integrated into routine clinical practice. This information provides a positive outlook for the uptake of newer targeted therapies in the second- and third-line settings. The rapid uptake of anaplastic lymphoma kinase (ALK) inhibitors such as crizotinib and ceritinib in patients with advanced NSCLC harboring *ALK* mutations provides further evidence that biomarker-directed therapy is an important treatment option for these patients [36].

Biomarker testing may impact uptake of newly approved targeted therapies. More information on how and when biomarker testing is performed in routine clinical practice is needed, since it is unclear in the included studies whether biomarker testing was more commonly performed prior to first-line versus second-line treatment. Across the European studies, erlotinib use increased despite approval of *EGFR* mutation testing in Europe after erlotinib approval. This suggests a trend among clinicians of using new therapies when clinically appropriate, despite limitations in access to biomarker testing. These trends imply positive uptake of new immunotherapies like programmed death-ligand 1/programmed death 1 (PD-L1/PD-1) checkpoint inhibitors despite evolving biomarker testing guidelines.

A few limitations should be considered when interpreting the information provided in this review. A meta-analysis could not be conducted due to the heterogeneity of the study designs and data presentation in the included studies. There were substantial differences in patient selection procedures, data collection methods, and outcome measure definitions. Thus, a qualitative data synthesis was the most appropriate way to summarize these studies [37]. Moreover, the review was robust and systematic, which renders the search and presentation of results transparent and reproducible. Additionally, publication bias could influence the evidence presented in this review; however, studies were found from multiple countries. Finally, conclusions from the results presented herein are limited by the observational design of the included studies.

## Conclusions

Real-world studies of second- and third-line treatment patterns in advanced NSCLC provide insights into how evidence from clinical trials impacts clinical practice. The studies included in this qualitative systematic review demonstrate the limited clinical benefit of the second- and third-line treatments for advanced NSCLC that were available at the time these studies were conducted. Within recent years, several novel therapies have been approved for use in these settings, and it will be important to determine the benefit that these and other new treatments may provide to patients with advanced NSCLC.

## Supporting information

S1 Table Search string. This table contains the search string that was used to perform the systematic review.
(DOCX)

S2 Table Assessment of bias. This table contains the assessment of bias for each study.
(DOCX)

S3 Table Summary of third-line treatment patterns. This table contains the summary of third-line treatments by country.
(DOCX)

S1 File PRISMA checklist. This document contains a completed PRISMA checklist.
(DOC)
