# Supplementary material for: Real-world treatment patterns for patients receiving second-line and third-line treatment for advanced non-small cell lung cancer: A systematic review of recently published studies
Source: PLoS One. 2017 Apr 14;12(4):e0175679. doi: 10.1371/journal.pone.0175679 (PMC5391942; doi:10.1371/journal.pone.0175679)
Supplement: S1 Table — This table contains the search string that was used to perform the systematic review. (DOCX) [file pone.0175679.s001.docx]

**S1 Table. Search string.**

| **Type of Search** | **Search String** |
| --- | --- |
| Standard indication search string (A) | ((MESH("Lung Neoplasms")) OR (MESH(“Carcinoma, Non-Small Cell Lung”)) OR (MESH(Adenocarcinoma, “Bronchiolo-Alveolar”)) OR (MESH(“drug therapy”)) OR (AB,TI(“lung tumour”)) OR (AB,TI(“lung carcinoma”)) OR (AB,TI(lung and squamous cell carcinoma)) OR (AB,TI(lung AND adenocarcinoma)) OR (AB,TI(lung AND “large cell carcinoma”)) OR (AB,TI(“non-small cell lung cancer”)) OR (AB,TI(“advanced nsclc”)) OR (AB,TI(“advanced non-small-cell lung”)) OR (AB,TI(“metastatic nsclc”)) OR (AB,TI(“metastatic non-small-cell lung”)) OR (AB,TI(metastatic non-small cell lung)) OR (((AB,TI(lung)) OR (AB,TI(nsclc)) OR (AB,TI(“non-small cell lung”))) AND ((AB,TI(“stage III”)) OR (AB,TI(“stage IV”)) OR (MESH(“lung neoplasms” “drug therapy”)) OR (MESH(Carcinoma “non-small cell lung” “drug therapy”))))) |
| Study methodology (B) | (MESH(observational) OR (AB,TI(observational)) OR AB,TI(“chart review”) OR (AB,TI(“retrospective study”)) OR (AB,TI(“prospective study”)) OR (AB,TI(“expanded access program”))) |
| Treatment patterns (C) | ((AB,TI(“second line”)) OR (AB,TI(relaps*)) OR (AB,TI(refract*)) OR (AB,TI(recurrent)) OR (AB,TI(resis*)) OR (AB,TI(“prior treatment”)) OR (AB,TI(“prior therapy”)) OR (AB,TI(“previously treated”)) OR (AB,TI(pretreated)) OR (AB,TI(“pre treated”)) OR (AB,TI(“pre-treated”)) OR (AB,TI(“rescue chemotherapy”)) OR (AB,TI(“failed chemotherapy”)) OR (AB,TI(progressive))) OR ((AB,TI(“second line”)) OR (AB,TI(advan*)) OR (AB,TI(“stage III”)) OR (AB,TI(“stage IV”))) AND ((AB,TI(docetaxel)) OR (AB,TI(paclitaxel)) OR (AB,TI(gemcitabine)) OR (AB,TI(vinorelbine)) OR (AB,TI(pemetrexed)) OR (AB,TI(erlotinib)) OR (AB,TI(EGFR-TKIs)) OR (MESH(platinum)) OR (AB,TI(“treatment pattern”)) OR (AB,TI(“cancer chemotherapy”)) OR (AB,TI(“targeted therapy”))) |
| Treatment outcome (D) | (AB,TI(survival)) OR (AB,TI(“response status”)) OR (AB,TI(“treatment outcome”)) |
