# Supplementary material for: Real-world treatment patterns for patients receiving second-line and third-line treatment for advanced non-small cell lung cancer: A systematic review of recently published studies
Source: PLoS One. 2017 Apr 14;12(4):e0175679. doi: 10.1371/journal.pone.0175679 (PMC5391942; doi:10.1371/journal.pone.0175679)
Supplement: S2 Table — This table contains the assessment of bias for each study. (DOCX) [file pone.0175679.s002.docx]

**S2 Table. Assessment of bias.**

| **Questions to Assess Risk of Bias Within Each Individual Study** | **Type of Bias Assessed** | **Younes et al, 2011** | **Sacher et al, 2015** | **Bischoff et al, 2010 (ACTION)** | **Moro-Sibilot et al, 2010; Vergnenegre et al, 2012 (SELECTIONN)** | **Carpentier et al., 2016** | **Zietemann, 2011; Zietemann, 2010** | **Reinmuth et al, 2013** | **Gridelli et al, 2014; De Marinis et al, 2014 (LIFE)** | **Gridelli et al, 2011 (SUN)** | **Asahina et al, 2012** | **Pan et al, 2013** | **Davis et al, 2015** |
| --- | --- | --- | --- | --- | --- | --- | --- | --- | --- | --- | --- | --- | --- |
| Do the inclusion/exclusion criteria vary across the comparison groups of the study? | Selection bias | No | No | No | No | No | No | No | No | No | No | No | No |
| Does the strategy for recruiting the participants into the study differ across groups? | Selection bias, confounding | No | No | No | No | No | No | No | No | No | No | No | No |
| Is the selection of the comparison group inappropriate, after taking into account feasibility and ethical considerations? | Selection bias, confounding | No description of multivariate methods used to select predictors of OS | Cox proportional hazards model with defined *P*-value threshold (*P* < .25) from univariate analysis for inclusion of factors into analysis | Cox proportional hazards model with defined *P*-value threshold (*P* < .10) from univariate analysis for inclusion of factors into analysis | Propensity scores calculated to assess cohort comparability; due to observed differences in patient characteristics associated with treatment choice, formal comparisons were not performed | Multiple regression analyses using Cox proportional hazard models. | Cox proportional hazards model with defined *P*-value threshold (*P* < .10) from univariate analysis for inclusion of factors into analysis | Cox proportional hazards model with defined *P*-value threshold (*P* < .10) from univariate analysis for inclusion of factors into analysis | Univariate and bivariate analyses only; study intent is descriptive | Univariate and bivariate analyses only; study intent is descriptive | Cox proportional hazards model with all pre-selected variables entered in a single step | Cox proportional hazards model with defined *P-*value threshold (*P* < .25) from univariate analysis for inclusion of factors into analysis plus selected key clinical variables | Multivariate logistic regression performed; separate models created to refine comparison groups |
| Does the study fail to account for important variations in the execution of the study from the proposed protocol? | Performance bias | No variations reported | No variations reported | Yes: study did not reach target sample size for recruitment despite extension of enrollment period from 6 to 18 months | No variations reported | No variations reported | No variations reported | No variations reported | No variations reported | No variations reported | No variations reported | No variations reported | No variations reported |
| Were valid and reliable measures, implemented consistently across all study participants used to assess inclusion/exclusion criteria, intervention/exposure outcomes, participant health benefits and harms, and confounding? | Detection bias, confounding | Selection criteria and outcomes were clearly defined | Selection criteria and outcomes were clearly defined | Selection criteria, study measures, and outcomes were clearly defined | Selection criteria and outcomes were clearly defined | Selection criteria and outcomes were clearly defined | Selection criteria and outcomes were clearly defined | Selection criteria and outcomes were clearly defined | Selection criteria and outcomes were clearly defined | Selection criteria and outcomes were clearly defined | Selection criteria and outcomes were clearly defined | Selection criteria and outcomes were clearly defined; progression measured via proxy algorithm—may have been underestimated | Selection criteria and outcomes were clearly defined |
| Was the length of follow-up different across study groups? | Attrition bias | Median follow-up: 9.1 months (0–108 months) | Median follow-up: 16.9 months (0.03–72.6 months) | Study does not report median follow-up time | Study does not report median follow-up time | Study does not report median follow-up time | Study does not report median follow-up time | Study does not report median follow-up time | 6-month follow-up period planned for all patients; study does not report median follow-up time | 12-month follow-up period planned for all patients; study does not report median follow-up time | Study does not report median follow-up time | Study does not report median follow-up time | Median follow-up: 6 months (0–121 months) |
| In cases of missing data (eg, overall or differential loss to follow-up) was the impact not assessed (eg, through sensitivity analysis or other adjustment method)? | Attrition bias, detection bias | Kaplan-Meier survival analysis | Kaplan-Meier survival analysis | Kaplan-Meier survival analysis; no information on number of patients lost to follow-up | Study accounted for loss to follow-up in time to treatment discontinuation analysis; missing data were not imputed | Kaplan-Meier survival analysis | Kaplan-Meier survival analysis | Kaplan-Meier survival analysis | Missing data were not imputed | Kaplan-Meier survival analysis | Kaplan-Meier survival analysis | Kaplan-Meier survival analysis | Oral medications were not included because the study did not use outpatient prescription data |
| Are any important primary outcomes missing from the results? | Selective outcome reporting | No | No | No | No | No | No | No | No survival outcomes reported—study objectives did not include survival analyses | No | No | No | No |
| Are any important harms or adverse events that may be a consequence of the intervention/exposure missing from the results? | Selective outcome reporting | Toxicity data were not evaluated as part of the study | Toxicity data were not evaluated as part of the study | Toxicity data were not evaluated as part of the study | No | Toxicity data were not evaluated as part of the study | Toxicity data were not evaluated as part of the study | No | No | Toxicity data were not reported as part of the study | Toxicity data were not reported as part of the study | Toxicity data were not reported as part of the study | Toxicity data were not reported as part of the study |
| Were the important confounding variables taken into account in the design and/or analysis (eg, through matching, stratification, interaction terms, multivariate analysis, or other statistical adjustment such as instrumental variables)? | Confounding | Study covers a long time (1990–2008) during which new NSCLC treatments were introduced. No information on whether treatment patterns changed over time, although a non-significant increase in OS was observed for years 1990–2003 vs 2004–2008 | Information on oral therapies, comorbid conditions, and performance status was not available from the databases used for this study | Study does not report histology beyond NSCLC; Cluster analysis performed to analyze survival within clinically similar patient sub-groups | Yes—propensity scores used to assess cohort comparability | Study covers a long time (1998-2005) and accounts for period differences. Additionally, multivariate analyses for prognostic factors were used | Yes - multiple logistic regression analysis | Study provides limited information on details of systemic therapy regimens; multivariate analyses performed using Cox regression model | No—study designed as a descriptive analysis | No—study designed as a descriptive analysis | Cox proportional hazards model used for multivariate analysis | Possible impact of unmeasured confounders—comorbidities and use of oral therapies were not fully captured in EHR database | Use of oral therapies not captured in this analysis; multivariate logistic regression performed |
| Are the results believable taking study limitations into consideration? | Overall assessment | Yes | Yes | Yes | Yes | Yes | Yes | Yes | Yes | Yes | Yes | Yes | Yes |

Abbreviations: EHR, electronic health record; NSCLC, non-small cell lung cancer; OS, overall survival.
