# Supplementary material for: Real-world treatment patterns for patients receiving second-line and third-line treatment for advanced non-small cell lung cancer: A systematic review of recently published studies
Source: PLoS One. 2017 Apr 14;12(4):e0175679. doi: 10.1371/journal.pone.0175679 (PMC5391942; doi:10.1371/journal.pone.0175679)
Supplement: S3 Table — This table contains the summary of third-line treatments by country. (DOCX) [file pone.0175679.s003.docx]

**S3 Table.** **Summary of third-line treatment patterns.**

| **Country** | **Reference** | **Number of Patients Enrolled in Study** | **Patients With Third-Line Treatment, n (%)^a^** | **Overall Third- Line Treatment Regimen Distribution, n (%)^b^** | **Distribution of Single-Agent Treatments, n (%)^b^** | **Distribution of Combination Regimens, n (%)^b^** | **Distribution of Targeted Therapy, n (%)^b^** |
| --- | --- | --- | --- | --- | --- | --- | --- |
| Brazil | Younes et al, 2011 | 2673 | 214 (8.01%) | NR | NR | NR | NR |
| Europe^c^ | Moro-Sibilot et al, 2010 Vergnenegre et al, 2012 (SELECTTION) | 1013 | 334 (33%) | NR | NR | NR | NR |
| France | Carpentier et al., 2016 | 1047 | 226 (21.6%) | Single agent 113 (50%)  Combination regimen 28 (12%)  Targeted therapy 85 (38%) | Docetaxel 18 (8%)  Pemetrexed 18 (8%)  Other 77 (34%) | Cisplatin based 11 (5%)  Carboplatin based 17 (8%) | Erlotinib or Gefitinib 83 (37%)  Other 2 (1%) |
| Germany | Zietemann, 2011; Zietemann, 2010 | 406 | 110 (27.1%) | Single agent 50 (47%) Targeted therapy 44 (41%) Other 13 (12%) | Docetaxel 22 (21%) Gemcitabine 14 (13%) Vinorelbine 8 (7%) Pemetrexed 6 (6%) | NA | Erlotinib 30 (28%) Gefitinib 14 (13%) |
| Germany^d^ | Reinmuth et al, 2013 | 493 | 97 (28%) | Single agent 49 (51%) Combination regimen 7 (7%) Targeted therapy 36 (37%) Other 5 (5%) | NR | Platinum based 7 (7%) | EGFR-TKI 36 (37%) |
| Italy^e^ | De Marinis et al, 2014;  Gridelli et al, 2014 (LIFE) | 541 | 158 (29%) | Single agent 83 (53%) Combination regimen 10 (6%) Targeted therapy 64 (41%) | Vinorelbine 32 (20%) Docetaxel 25 (16%) Gemcitabine 16 (10%) Pemetrexed 6 (4%) Paclitaxel 3 (2%) Topotecan 1 (1%) | Gemcitabine + vinorelbine 3 (2%) Carboplatin + Gemcitabine 2 (1%) Carboplatin + pemetrexed 1 (1%) Carboplatin + vinorelbine 1 (1%) Docetaxel + gemcitabine 1 (1%) Docetaxel + vinorelbine 1 (1%) Cisplatin + gemcitabine 1 (1%) | Erlotinib 64 (40%) Other 1 (1%) |
| Italy^f^ | Gridelli et al, 2011 | 987 | 58 (7%) | Single agent 30 (52%) Combination regimen 7 (12%) Targeted therapy 21 (36%) | Pemetrexed 11 (19%) Docetaxel 6 (10%) Vinorelbine 6 (10%) Gemcitabine 4 (7%) Paclitaxel 2 (3%) Other 1 (2%) | NR | Erlotinib 21 (36%) |
| Japan^g^ | Asahina et al, 2012 | 599 | 230 (38%) | Single agent 118 (51%) Targeted therapy 69 (30%) Investigational new drug 25 (11%) Other 18 (8%) | Docetaxel 80 (35%) S-1 28 (12%) Gemcitabine 10 (4%) | NA | Gefitinib 69 (30%) |
| United States^h^ | Pan et al, 2013 | 1168 | NR | NR | NR | NR | NR |
| United States^i^ | Davis et al, 2015 | 17,133 | 1713 (24%) | Single agent 845 (49%) Combination regimen 618 (36%) Other 250 (15%) | Gemcitabine 280 (16%) Pemetrexed 209 (12%) Docetaxel 171 (10%) Vinorelbine 117 (7%) Paclitaxel 68 (4%) | Carboplatin + gemcitabine 238 (14%) Carboplatin + paclitaxel 229 (13%) Carboplatin + docetaxel 69 (4%) Gemcitabine + vinorelbine 44 (3%) Cisplatin + gemcitabine 38 (2%) | NR |

Abbreviations: EGFR-TKI, epidermal growth factor receptor tyrosine kinase inhibitor; NA, not applicable; NR, not reported; NSCLC, non-small cell lung cancer.

^a^Percentage of study cohort.

^b^Percentage of patients with third-line treatment.

^c^Proportion of patients treated with first-line regimens were estimated from graph in Appendix 3 of Moro-Sibilot et al 2010 [18].

^d^Only consisted of patients with stage IV NSCLC.

^e^10% of patients were excluded after enrollment due to not meeting inclusion criteria (evaluable patients N = 541).

^f^80% (N = 790) of the original cohort (987) were followed due to 12% being enrolled in clinical trials in the first line and 9% only receiving best supportive care.

^g^Study investigators only reported top 5 regimens for third line.

^h^Only included patients who received planned second-line treatment and had non-squamous histology.

^i^Only included patients who had squamous histology.

Note: The following studies did not report complete information on third-line treatment:

1. Canada: Sacher et al, 2015 [20]

Europe: Bischoff et al, 2010 [14]
